# Distorted Taste and Impaired Oral Health in Patients with Sicca Complaints

**DOI:** 10.3390/nu11020264

**Published:** 2019-01-24

**Authors:** Preet Bano Singh, Alix Young, Amin Homayouni, Lene Hystad Hove, Beáta Éva Petrovski, Bente Brokstad Herlofson, Øyvind Palm, Morten Rykke, Janicke Liaaen Jensen

**Affiliations:** 1 Department of Oral Surgery and Oral Medicine, Faculty of Dentistry, University of Oslo, 0317 Oslo, Norway; b.b.herlofson@odont.uio.no (B.B.H.); j.c.l.jensen@odont.uio.no (J.L.J.); 2 Department of Cariology and Gerodontology, Faculty of Dentistry, University of Oslo, 0317 Oslo, Norway; a.y.vik@odont.uio.no (A.Y.); amin.homayouni@odont.uio.no (A.H.); l.h.hove@odont.uio.no (L.H.H.); morten.rykke@odont.uio.no (M.R.); 3 Dentistry Administration, Faculty of Dentistry, University of Oslo, 0317 Oslo, Norway; beata.petrovski@odont.uio.no; 4 Department of Rheumatology, Oslo University Hospital, 0317 Oslo, Norway; oypalm@gmail.com

**Keywords:** taste, smell, dysgeusia, burning sensation, halitosis, saliva, caries, primary Sjögren’s syndrome, non-SS sicca syndrome

## Abstract

Senses of smell and taste, saliva flow, and dental status are considered as important factors for the maintenance of a good nutritional status. Salivary secretory rates, chemosensory function, burning mouth sensation, halitosis and dental status were investigated in 58 patients with primary Sjögren’s syndrome (pSS), 22 non-Sjögren’s syndrome sicca (non-SS) patients, and 57 age-matched healthy controls. A significantly greater proportion of patients with pSS and non-SS had ageusia, dysgeusia, burning mouth sensation, and halitosis compared to controls. Patients with pSS had significantly lower olfactory and gustatory scores, and significantly higher caries experience compared to controls. Patients with pSS and non-SS patients had significantly lower unstimulated and stimulated whole saliva secretory rates compared to controls. The findings indicated that several different aspects of oral health were compromised in both, patients with pSS and non-SS, and this may affect their food intake and, hence, their nutritional status. Although non-SS patients do not fulfill Sjögren’s syndrome classification criteria, they have similar or, in some cases, even worse oral complaints than the patients with pSS. Further studies are needed to investigate food preferences, dietary intake, and nutritional status in these two patient groups in relation to their health condition.

## 1. Introduction

Nutritional status is closely associated with health status, and decline in dietary intake can lead to weight loss and increased risk for disease [1]. The senses of smell and taste are important for nutrition—smell is vital in identifying potential dietary substances in the environment, while taste is instrumental in voluntary ingestion and early digestion of these dietary substances [2]. Saliva and nasal mucus are important for maintaining normal function of the taste buds imbedded in the oral epithelium and olfactory cells found in the nasal cavity [3]. Patients with reduced salivary secretion are known to have taste and smell abnormalities [3,4]. Furthermore, nutritional status is impaired in patients with taste and smell disorders [5]. Most studies showing taste and smell abnormalities in patients with dry mouth are reported from patients with Sjögren’s syndrome. Little is known about patients having similar symptoms of severe dry mouth and dry eyes, but not fulfilling the classification criteria for Sjögren’s syndrome.

Sjögren’s syndrome (SS) is an autoimmune connective tissue disorder of the exocrine glands, primarily the salivary and lacrimal glands [6]. A long-lasting inflammatory process in glandular tissue can lead to the loss of glandular cells, resulting in reduction or, in the worst cases, even complete loss of saliva and tear secretions [7]. The disorder has an unknown etiology, and mainly affects women [8]. The female to male ratio has been reported to be nine to one [8].

To be classified for SS diagnosis, patients have to fulfill at least four out of six classification criteria [9]. These criteria include symptoms of dry mouth and dry eyes; reduced tear secretion; reduced saliva secretion; histopathology of minor salivary glands showing infiltrates of lymphocytes; and the presence of autoantibodies directed against Ro/SSA (anti-Sjögren’s-syndrome-related antigen A, also called anti-Ro) and/or La/SSB (anti-Sjögren’s-syndrome-related antigen B, also called anti-La) [9]. As long as either serological or histopathological tests are positive, the presence of any four out of six symptoms indicates SS. If three out of four objective symptoms are present, it also justifies classifying the patient with SS. Patients complaining of dry eyes and dry mouth, but not fulfilling all the required criteria, are referred to as non-Sjögren’s syndrome sicca (non-SS) patients.

Sjögren’s syndrome can be subdivided into primary and secondary Sjögren’s syndrome. Primary Sjögren’s syndrome (pSS) is a diagnosis given to patients with manifest symptoms of dryness in the absence of other connective tissue diseases. Secondary Sjögren’s syndrome (sSS) describes patients with symptoms of dryness, in the setting of another connective tissue disease or chronic inflammatory process, such as rheumatoid arthritis, systemic lupus erythematosus, diagnosed prior to developing SS symptoms [10]. The prevalence of pSS has been reported to range from 0.03% to 2.7% worldwide when different classification criteria were applied [11]. When applying the criteria of the American–European Consensus Group, the prevalence of pSS in the Norwegian population is estimated at 0.05% [12].

Patients with pSS and non-SS display a wide range of similar symptoms; among these are xerostomia—the subjective sensation of oral dryness. Symptoms of dry mouth often include frequent feeling of thirst, feeling of dryness in the mouth and throat, and ulcers may occur in the oral cavity [13]. Patients with dry mouth often have problems with decreased taste sensitivity and chewing in addition to difficulties with articulation [14]. Although, patients categorized as non-SS have similar complaints as patients with pSS, there is a risk that they do not receive appropriate medical care by the health authorities because of lacking diagnosis of SS.

Olfactory and gustatory disorders, also known as chemosensory disorders, are the disorders affecting the senses of smell and taste. Chemosensory disorders are categorized into quantitative and qualitative disorders, depending on whether the senses are reduced or distorted, respectively. Following this categorization, olfactory disorders are classified into anosmia (complete loss of smell), hyposmia (reduced ability to smell), and dysosmia (distorted sense of smell) [15]. Similarly, gustatory disorders are classified as ageusia (complete loss of taste), hypogeusia (reduced ability to taste), and dysgeusia (distorted taste, for example, metallic taste perception) [16]. Patients with a normal sense of smell and taste are categorized as normosmic and normogeusic, respectively. Other oral disorders, like halitosis/oral malodor and burning sensation/numbness in the oral cavity, are often observed in patients with chemosensory disorders [4]. About 50% of patients with chemosensory disorders have reported a negative impact on (i) appetite and body weight, (ii) quality of life, and (iii) psychological well-being [17].

There is evidence that patients with SS have a poor dental status [18]. In a cross-sectional study of Chilean SS-patients, as many as 60% had dental caries, a higher prevalence than the general population [18]. However, in another study, no significant differences could be detected in the dental caries experience of Swedish SS patients compared to dry mouth controls [18,19]. Patients with pSS are also reported to have a significantly higher dental caries experience, also called DMFT (DMFT: decayed, missing, and filled teeth) than healthy controls, mainly due to a higher number of filled and missing teeth [20]. A change in a patient’s dental caries status has been suggested as one of several potential markers of the extent of autoimmune-mediated salivary gland dysfunction in pSS [20].

The aim of this study was to compare salivary flow, olfactory and gustatory function, burning mouth sensation, halitosis, and dental status in patients with pSS, non-SS sicca patients, and healthy age-matched controls, to gain more insight into the oral status of non-SS sicca patients.

## 2. Materials and Methods 

### 2.1. Participants

The study was conducted at the Dry Mouth Clinic at the University of Oslo (UiO), Norway, and was approved by the Norwegian Regional Committee for Research Ethics (REK 2015/363). Another study has previously been published with the same REK number which includes 31 female patients with pSS and 33 gender-matched controls [4]. The present study presents an additional patient group (22 non-SS patients), and a higher number of patients, in both pSS group (58 patients with pSS) and healthy control group (57 healthy controls). Moreover, different parameters have been investigated in the two studies. The data presented in this study has not been published before. Participant characteristics are presented in Table 1. Written informed consent was obtained from all participants prior to examination. Most patients with pSS were referred from the Department of Rheumatology at Oslo University Hospital (OUS), where they were classified according to the American–European Consensus Group criteria (13). Non-SS patients were referred to the last author J.L.J for salivary gland biopsies [13]. They all had sicca complaints, but anti-Ro/SSA were absent, and the histopathology of their salivary gland biopsies were not consistent with pSS. The exclusion criteria for controls were mouth and eye dryness, chronic diseases, and use of medications that could affect the salivary glands. The participants were instructed to refrain from eating, drinking, and smoking one hour prior to examination. The assessments of salivary secretory rates, olfaction, gustation, oral malodor, and dental status were carried out by a team of calibrated dental practitioners and specialists.

### 2.2. Saliva Assessment 

Summated Xerostomia Inventory-Dutch (SXI-D) version was used to assess participants’ self-reported perception of dry mouth [21]. SXI-D is a shortened version of the Xerostomia Inventory (XI) [22] and consists of five statements that are used to determine the severity of xerostomia. The SXI-D sum score ranges from 5 to 15, where 15 = very severe problems related to xerostomia. Thereafter, unstimulated (UWS) and chewing-stimulated (SWS) whole saliva were collected from all participants to determine salivary secretory rates. Unstimulated whole saliva was collected first for 15 min, and then SWS for 5 min. Saliva samples were weighed and secretory rates were calculated for UWS and SWS (g/min = mL/min). UWS secretory rate was considered normal if ≥ 0.1mL/min, and SWS secretion rate was considered normal if ≥ 0.7 mL/min [23].

### 2.3. Olfactory Assessment

Self-reported perception of sense of smell was obtained prior to olfactory testing. Participants were asked to score their own subjective smell perception on a visual analogue scale (VAS) from 0 to 10, where 0 = no smell perception, and 10 = very good smell perception. Cognitive olfactory function was measured using twelve-stick identification test (Burghart Messtechnik, Wedel, Germany). The participants were instructed to choose from four possible answers on a multiple choice-scoring card. The answers were recorded, and the data were summarized for each participant. A normative classification [24] was used to define anosmic (score 0–5), hyposmic (score 6–9), and normosmic (score 10–12) participants.

### 2.4. Gustatory Assessment

Self-reported perception of sense of taste was obtained prior to gustatory testing. Participants were asked to score their own subjective taste perception on a visual analogue scale (VAS) from 0 to 10, where 0 = no taste perception, and 10 = very good sense of taste. Gustatory function was evaluated using taste strips (Burghart Messtechnik, Wedel, Germany) with four basic taste qualities; sweet, sour, salty, and bitter, each tested at 4 different concentrations. The taste qualities were presented in a random manner, starting with the weakest concentrations. This protocol resulted in a total of 32 values for each participant, as both sides of the tongue were tested. A normative classification [25] was followed to distinguish between ageusic (score 0–12), hypogeusic (score 13–18), and normogeusic (score 19–32) participants.

### 2.5. Assessment of Dysgeusia, Burning Mouth Sensation, and Halitosis

A questionnaire was designed for use in this study to assess participants’ experience of dysgeusia, burning mouth sensation (BMS), and halitosis (Table 2). The present questionnaire is a modified version of a questionnaire that we have published in a previous study [4]. Both patients with pSS and non-SS reported that they had periods when their disease symptoms were more pronounced (“bad periods”) and periods when the symptoms were less pronounced (“good periods”).

### 2.6. Oral Malodor Assessment

Self-reported perception of halitosis was obtained prior to oral gas sampling. Participants were asked to score their own subjective perception of oral malodor on a scale from 0 to 5, where 0 = no appreciable odor, and 5 = extremely foul odor. Halitosis was measured using both organoleptic and objective methods. The organoleptic measurements were performed by instructing the participants to exhale briefly through the mouth at three different distances (100, 30, and 10 cm) from the nose of the organoleptic judge. The level of malodor was recorded using the same scale as for the self-reported perception of halitosis [26]. Levels of volatile sulfur compounds (VSC) in the mouth air of the participants were measured by gas chromatography (GC: OralChroma™, Nissha FIS, Inc., Osaka, Japan). Mouth air samples from the participants were obtained using a standardized procedure according to the user manual. A 1.0 mL syringe was inserted into the oral cavity until the stopper was in contact with the lips and the syringe could be held gently between the teeth without the tongue touching the tip of the syringe. After the syringe was held in this position for 30 s, a mouth air sample was withdrawn using the syringe, and was immediately injected into the OralChroma™. Analysis of VSC started automatically, and the levels of hydrogen sulfide (H_2_S) and methyl mercaptan (CH_3_SH) determined. The olfactory threshold levels (in parts per billion, ppb) indicating oral malodor were considered either high (H_2_S > 112 ppb and CH_3_SH > 26 ppb) or low (H_2_S < 112 ppb and CH_3_SH < 26 ppb), as recommended by the manufacturer and used in other studies [27].

### 2.7. Dental Assessment 

Self-reported perception of dental health and general health was obtained from the participants prior to clinical and radiological examination of the teeth. Participants were asked to score their own subjective assessment of their dental and general health status on a scale from 0 to 5, where 0 = very poor, and 5 = excellent. A thorough dental examination, consisting of clinical and radiological examination of the oral cavity, was conducted by general dental practitioners. The number of decayed, missing, or filled teeth (DMFT) and only filled teeth (FT) were recorded [23].

### 2.8. Statistical Analyses 

Descriptive statistical analysis was performed, and the results are presented in percentages, median/interquartile range (IQR)/ranges. Normality of continuous variables was tested on histogram, Q–Q plot, and by Shapiro–Wilk test. Due to the low sample size and non-normal distribution of the continuous variables, Kruskal–Wallis ANOVA and Mann–Whitney *U* test was used to detect median differences of continuous, numerical variables between the two or three groups (control, non-SS, pSS). Chi-square (χ2) test and Fischer’s-exact test was used to test the differences of the distribution of categorical variables. Point-biserial and Spearman correlations were used to measure the strength and direction of the association between the one continuous and one dichotomous variable, and between two continuous variables respectively. All differences were considered significant at *p* < 0.05. Statistical Package for STATA (Stata version 14.0; College Station, TX, USA) and SPSS (SPSS version 24, IBM, Armonk, NY, USA) were used for the statistical analyses.

## 3. Results

### 3.1. Dysgeusia, Burning Mouth Sensation, and Halitosis

Self-reported complaints of dysgeusia, burning mouth sensation, and halitosis in the three groups are shown in Table 3. The completion rate for Yes/No questions in the questionnaire was 100% in the three groups. The frequency of dysgeusia, burning sensation, and halitosis was significantly higher in the non-SS and pSS groups versus controls, and these self-reported complaints showed significant association with the disease (*p* < 0.001).

Fifteen patients with non-SS, thirty-one patients with pSS and one participant in the control group, who experienced dysgeusia, answered further questions. Metallic taste dysgeusia was the most common complaint both in the non-SS and pSS groups. Other taste distortions were described as “rotten” and “bitter”, in addition to “other” taste distortions which the participants were not able to describe in words. Distorted taste was significantly more common in the non-SS and pSS groups, compared to controls (*p* < 0.001) (Table 4).

Some patients with pSS and non-SS described that they had good and bad periods, where the disease symptoms were less pronounced in good periods and more pronounced in bad periods. The duration of good and bad periods varied between individuals. Dysgeusia was experienced either “constantly”, “daily”, “sometimes, or “in bad periods”. The perceived distorted taste was significantly more frequent in the non-SS and pSS groups, compared to controls (*p* < 0.001) (Table 5).

When answering the question “is bad taste related to meals?”, some participants reported dysgeusia “during meals”, others experienced it “in between meals”, while some reported “constant” lingering of bad taste in the mouth. Only patients with pSS reported that bad taste was more pronounced “during meals”, resulting in foul-tasting meals. One of the patients with pSS reported that even water had a metallic taste. Moreover, “constant” perception of dysgeusia was also found only in the pSS group (Table 6).

The response rate for the multiple choice questions for burning mouth sensation among the participants experiencing burning mouth, was almost 30% in the non-SS group, 65% in the pSS group and 50.0% controls. Majority of these participants experienced a burning sensation on the “whole tongue”, while some patients experienced this only on the “anterior tongue”. Only one patient with pSS experienced a burning sensation on the “lips and palate” in addition to the tongue. A significantly higher proportion of participants experienced burning mouth sensation on the tongue, compared to controls (*p* < 0.001) (Table 7). An overview of how often participants in the three groups experienced burning mouth sensation is shown in Table 8.

For some of the patients complaining of burning mouth sensation, it was reported to be worst “during meals” in the non-SS and pSS groups (Table 9). Twenty-seven percent of non-SS patients and 24% of patients with pSS reported that they had to refrain from food items like spicy food, sour food items, sour fruits, and beverages like soft drinks, juices, and wine, because of burning mouth sensation.

Among participants complaining of halitosis, non-SS patients were more affected than patients with pSS, while none of controls complained of oral malodor. Table 10 shows how often participants experienced oral malodor. Some patients reported that they avoided drinking tea or coffee because of perceived risk of getting halitosis. When answering “which of the disturbances have a negative effect on your quality of life?”, both non-SS patients and patients with pSS reported burning mouth sensation and distorted taste as major factors affecting their quality of life.

### 3.2. Gustatory Function

**Figure 1:**
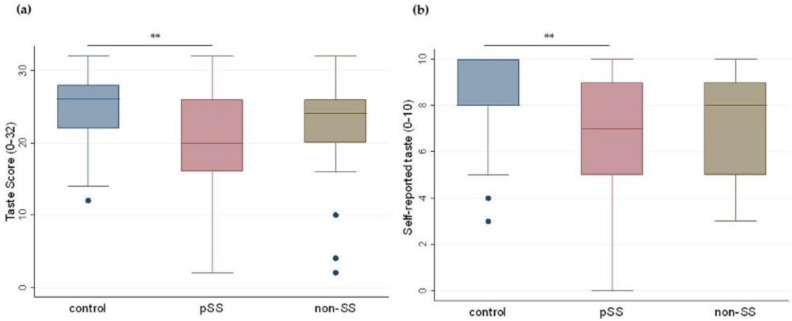
Measured and self-reported taste score in the three groups. Boxplots illustrating (**a**) measured taste scores and (**b**) participants’ self-reported taste score in controls, primary Sjögren’s patients (pSS), and non-Sjögren’s sicca patients (non-SS). (Kruskal–Wallis ANOVA and Mann–Whitney *U* test; ** *p* < 0.01.). Dots in the figures represent the outliers.

The results of the Kruskal–Wallis ANOVA and the Mann–Whitney *U* test showed that the measured median gustatory scores (median (IQR), range) were significantly lower in the pSS group (20.0 (16.0–26.0), 2.0–32.0) than in the control group (26.0 (22.0–28.0), 12.0–32.0) (*p* = 0.001). No significant differences were observed between the non-SS (24.0 (20.0–26.0), 2.0–32.0) and the control group (Figure 1a). Participants’ self-reported taste scores also revealed a significantly lower mean perception of taste in the pSS group (7.0 (5.0–9.0), 0.0–10.0) compared to the control group (8.0 (8.0–10.0), 3.0–10.0), (*p* = 0.009). No significant difference was found comparing the non-SS group (8.0 (5.0–9.0), 3.0–10.0) with controls (Figure 1b). Chi-square tests showed that a significantly higher percentage of pSS and non-SS patients had ageusia compared to controls (Table 11).

### 3.3. Olfactory Function

**Figure 2:**
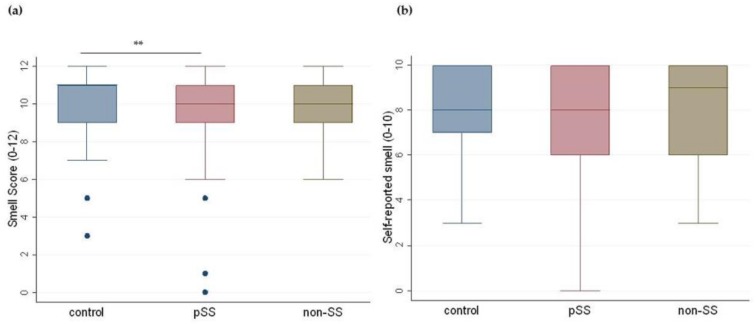
Measured and self-reported smell score in the three groups. Boxplots illustrating median, interquartile ranges (IQRs), and ranges of (**a**) measured smell scores (0–12) and (**b**) participants’ self-reported smell scores (0–10) in controls, primary Sjögren’s patients (pSS), and non-Sjögren’s sicca patients (non-SS). (Kruskal–Wallis ANOVA and Mann–Whitney *U* test; ** *p* < 0.01.). Dots in the figures represent the outliers.

The results of the Kruskal–Wallis ANOVA and the Mann–Whitney *U* test showed that the measured median olfactory scores (median (IQR), range) were significantly lower in the pSS group (10.0 (9.0–11.0), 0.0–12.0) than in the control group (11.0 (9.0–11.0), 3.0–12.0), (*p* = 0.007). No significant differences were observed between the non-SS (10.0 (9.0–11.0), 6.0–16.0) and the control group (Figure 2a). Participants’ self-reported smell scores did not reveal any significant differences between the three groups (Figure 2b).

### 3.4. Oral Malodor Results 

Gas chromatographic analysis (median (IQR), range) revealed the following H_2_S-values (ppb): control group (33.5 (8.7–141.0), 0.0–2885.0), pSS group (27.5 (15.7–96.2), 0.0–458.0), and non-SS group (41.0 (13.5–84.0), 0–803.0). The results for CH_3_SH (ppb) were as follows: control group (8.0 (3.0–26.5), 0–193.0), and for the pSS and non-SS groups were (6.0 (2.0–13.2), 0–75.0) and (5.0 (0–13.2), 0–83.0), respectively. There were no significant differences in H_2_S and CH_3_SH levels between the groups.

There was no significant correlation between the self-reported perception of halitosis and the organoleptic measurements. The self-reported perceived halitosis scores (median (IQR), range) for the control group were (0.0 (0.0–1.0), 0–3), while the scores for the pSS group and non-SS group were (1.0 (0.0–2.0), 0–4) and (2.0 (1.0–3.0), 0–5), respectively. Organoleptic judge scores were (0.0 (0.0–1.0), 0–2) for the control group, (0.0 (0.0–1.0), 0–3) for the pSS group, and (0.0 (0.0–1.0), 0–2) for the non-SS group. No significant differences were found between groups in self-reported perception of halitosis and organoleptic measurements.

### 3.5. Saliva and SXI-D

**Figure 3:**
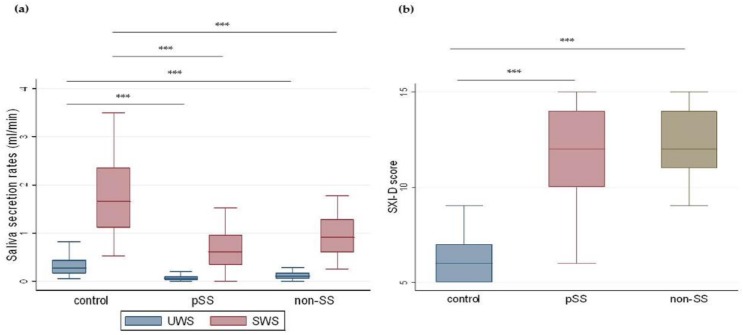
Measured saliva secretory rate and self-reported perception of xerostomia in the three groups. Boxplots illustrate median, IQRs, and ranges of (**a**) saliva secretion rates (mL/min) and (**b**) SXI-D: Summated Xerostomia Inventory-Dutch scores in controls, primary Sjögren’s patients (pSS), and non-Sjögren’s sicca patients (non-SS). (Kruskal–Wallis ANOVA and Mann–Whitney *U* test; *** *p* < 0.001.)

The results of the Kruskal–Wallis ANOVA and the Mann–Whitney *U* test showed that the UWS secretory rates (mL/min) were significantly lower in the pSS group (0.1 (0.0–0.1), 0.0–0.4) and non-SS group (0.1 (0.0–0.2), 0.0–0.6) compared to the control group (0.3 (0.2–0.4), 0.0–0.8), (*p* < 0.001) (Figure 3a). Also, SWS secretory rates were significantly lower in the pSS group (0.7 (0.4–1.0), 0.0–1.5) and non-SS group (0.9 (0.6–1.3), 0.3–1.8) compared to controls (1.6 (1.1–2.4), 0.5–3.5), (*p* < 0.001) (Figure 3a). The results of participants’ self-reported perception of xerostomia showed significantly higher SXI-D scores in both the pSS group (12.0 (10.0–14.0), 6.0–15.0) and the non-SS group (12.0 (11.0–14.0), 9.0–15.0) compared to controls (6.0 (5.0–7.0), 5.0–9.0), (*p* < 0.001) (Figure 3b). No significant differences were observed between pSS and non-SS groups, for either salivary secretory rates or SXI-D score.

Pathologically low saliva secretory rates for UWS (≤ 0.1 mL/min) and SWS (≤ 0.7 mL/min were analyzed among the participants. A significantly higher proportion of patients with pSS had saliva secretory level below the threshold level for both UWS and SWS (Table 12). Furthermore, moderate, significant correlations were found between salivary secretory values (USW and SWS) and dysgeusia, burning mouth sensation, halitosis, taste score, DMFT, and FT, when all the participants were considered together (Table 13).

### 3.6. DMFT/FT

**Figure 4:**
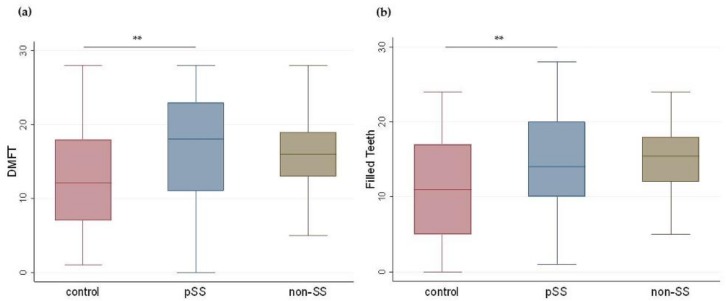
DMFT and FT results from the three groups. Boxplots illustrate median, IQRs, and ranges of (**a**) DMFT: decayed, missing, and filled tooth surfaces and (**b**) FT: filled teeth in controls, primary Sjögren’s patients (pSS), and non-Sjögren’s sicca patients (non-SS). (Kruskal–Wallis ANOVA and Mann–Whitney *U* test; ** *p* < 0.01.)

Caries experience as measured by DMFT (median (IQR), range) was significantly higher in the pSS group (18.0 (11.0–23.0), 0.0–28.0) compared to the control group (12.0 (6.5–18.0), 1.0–27.0), (*p* = 0.005). The DMFT in the non-SS group (16.0 (12.8–19.3), 0.0–28.0) did not differ from that of the control group (*p* = 0.3) or the pSS group (*p* = 1.0) (Figure 4a).

Similarly, the FT component of the DMFT index (median (IQR), range) was significantly higher in the pSS group (14.0 (10.0–20.0), 1.0–27.0) than the control group (11.0 (5.0–17.0), 0.0–24.0), (*p* = 0.030). The FT score in the non-SS group (15.5 (11.8–18.2), 0.0–24.0) did not differ from that of the control group (*p* = 0.241) or the pSS group (*p* = 1.0) (Figure 4b).

### 3.7. General Health Status and Dental Status

**Figure 5:**
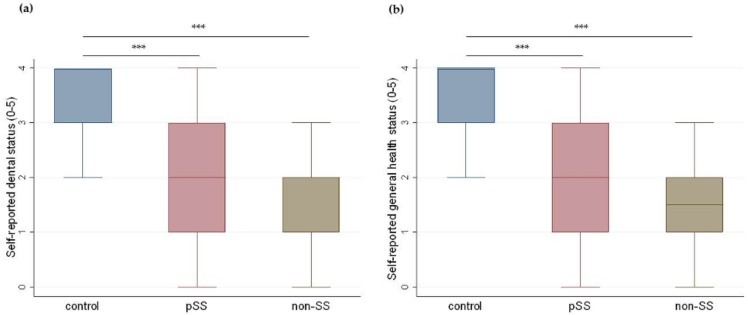
Self-reported dental and general health status in the three groups. Boxplots illustrate median, IQRs, and ranges of (**a**) self-reported dental status (0–5) and (**b**) self-reported general health status (0–5) in controls, primary Sjögren’s patients (pSS), and non-Sjögren’s sicca patients (non-SS). (Kruskal–Wallis ANOVA and Mann–Whitney *U* test; *** *p* < 0.001.)

Statistically significant differences were found in self-reported general health status (median (IQR), range) between patients with pSS (2.0 (1.0–3.0), 0–4.0), non-SS patients (1.5 (1.0–2.0), 0–3.0), and controls (4.0 (3.0–4.0), 2.0–4.0), *p* < 0.0001 (Figure 5b). Similar statistically significant differences were found between patients with pSS (2.0 (1.0–3.0), 0–4.0), non-SS patients (1.0 (1.0–2.0), 0–3.0), and controls (3.0 (3.0–4.0), 2.0–4.0), *p* < 0.0001, when participants scored their own dental health status (Figure 5a). Spearman’s test showed that when all participants were considered together, participants self-reported dental health status was found to be significantly, negatively correlated to dental status DMFT (*r* = −0.27, *p* = 0.001) and FT (*r* = −0.18, *p* = 0.04). Furthermore, significant positive correlations were found between participants’ dental and general health status (*r* = 0.58, *p* < 0.001).

## 4. Discussion

The present study revealed that the non-SS patients have similar or even worse oral health than patients with Sjögren’s syndrome. In general, patients with sicca symptoms, suspected to have SS but not fulfilling the classification criteria for SS, far outnumber the patients who fulfill the criteria. Still, only patients who fulfill the criteria are usually included in studies [28]. Thus, non-SS patients are left both without a diagnosis and are often not considered to be of interest for researchers. Therefore, the main focus in this study was the oral health status of the sicca patients without a Sjögren’s diagnosis.

In the present study, we found that complaints of dysgeusia, burning mouth sensation, and halitosis were common in the non-SS group. It has previously been shown that patients with pSS have a high percentage of complaints of dysgeusia, burning sensation on the tongue, and halitosis, and that about 50% of patients with pSS report these disorders [4]. In the present study, when comparing non-SS patients with patients with pSS, it was found that non-SS patients had a much higher occurrence of dysgeusia, burning mouth sensation, and halitosis. In the literature, there are no studies available to compare our current findings with results from other studies on non-SS patients. To our knowledge, this is the first study comprehensively evaluating oral health in patients with sicca symptoms without an SS diagnosis. Since many of the patients avoided certain food items due to problems with dysgeusia and burning sensations, it may affect their dietary intake. This is consistent with the literature, where decreased appetite has been reported in 30% of patients and decreased enjoyment of food in 70% of patients complaining of dysgeusia [29]. About 60% of dysgeusia patients have been reported to change their eating patterns and 40% to modify their use of seasonings [29].

In addition to distorted taste, reduced taste function was observed in both non-SS and patients with pSS. Ageusia, a condition characterizing complete absence of taste perception, is a very seldom condition and accounts for less than 1% of patients referred to taste and smell research centers [5,17]. When taste function was measured, almost 15% of non-SS and patients with pSS were found to have ageusia in this study. Loss of appetite has been reported in patients suffering from ageusia [5]. Furthermore, about 10% of non-SS patients and 26% of patients with pSS were found to be hypogeusic. The incidence of this taste disorder is also low in the general population, and some but not all patients suffering from hypogeusia report decreased enjoyment of food and decreased appetite [5]. However, hypogeusia in combination with other disorders, like dysgeusia and burning sensations, might exacerbate the changes in dietary intake [30]. Therefore, dietary intake monitoring and counselling is very important in those patients with pSS and non-SS patients that suffer from both qualitative and quantitative taste disorders.

Smell and taste disorders are common in the general population, however, patients are frustrated due to the lack of appropriate medical attention and care [17,31]. This may partly be a result of a lack of knowledge and focus on appropriate tools required to assess disorders involving chemical senses among medical practitioners. In this paper, we present a novel questionnaire that can be used to assess (i) patient’s chemosensory and trigeminal disorders, (ii) their duration, (iii) their effect on food preferences, and (iv) the effect on patient’s quality of life. This questionnaire may be helpful for nutritionists and other health professionals in getting an overview of patients’ oral disturbances. This will further be beneficial in managing patients’ dietary intake. The questionnaire consists of questions with yes and no answers, supplemented with multiple choice questions, and with the option “other”, for open-ended answers. It is easy to fill in and not time-consuming. Therefore, it is practical for use both in clinical and research settings. One of the limitations in the present study is that we did not attain a full rate of completion of the questionnaire, as it was first introduced when we realized that patients were having major issues with dietary intake due to their oral health complications. Further studies are needed to validate this questionnaire.

A large proportion of patients reported dietary limitations because of either dysgeusia, burning mouth sensation, halitosis, dry mouth, or a combination of these different oral problems. A synergetic relationship between oral health and nutrition has been suggested [32], in other words, the relationship may be considered as a positive feedback or a vicious circle. Oral conditions, caused by either local or systemic diseases, impact the functional ability to eat and vice versa, and decline in dietary intake can lead to progression of oral diseases [32]. However, little is known about dietary implications and oral disorders in patients with dry mouth symptoms without SS diagnosis. Further studies are needed to gain better insight into mechanisms leading to oral disorders in this group of patients.

In the present study, there were no significant differences in salivary secretory rates between the two patient groups, indicating that both patient groups have similar problems with dryness of the mouth. Results from self-reported mouth dryness scores and measured salivary secretory rates were also well correlated in this study, indicating severe mouth dryness. Furthermore, significant associations were found in this study, among participants with pathologically low salivary flow rates and oral disorders (chemosensory disturbances, trigeminal disorders, halitosis, and DMFT), consistent with other studies [3,33,34]. These oral disturbances can affect the integrity of the oral cavity and, hence, lead to malnutrition [32].

Patients with pSS had a significantly higher number of decayed, filled, or missing teeth compared to non-SS patients and controls. The dental treatments performed on patients included dental fillings, crowns, and bridges. The reason behind extensive dental treatment may be related to low salivary secretory rates, presence of oral disorders, and/or dietary preferences. Interestingly, non-SS patients share similar symptoms with patients with pSS regarding salivary flow rates and oral disorders, but the same degree of dental treatment was not observed in this group. Other systemic, inflammatory causes in SS may therefore be considered as a potential cause of high caries experience in patients with pSS. These findings are consistent with other studies where dental caries status has been suggested as one of several potential markers of the extent of autoimmune-mediated salivary gland dysfunction in pSS [20].

About 60% of non-SS patients and 40% of patients with pSS reported halitosis when answering the questionnaire. However, no significant differences were observed between these two groups regarding subjective and objective measurements of oral malodor. Halitosis experienced in these patients could therefore neither be confirmed by organoleptic assessment, nor by analysis of VSC levels measured by gas chromatography. The difficulty in the self-assessment of breath odor has been discussed by Rosenberg [35]. Furthermore, organoleptic assessments may assess foul-smelling gases other than those containing sulfur (VSC), and this may explain the difference between organoleptic scores and VSC levels measured by GC. These findings are consistent with other studies where clinicians have reported that one-third of the patients seeking treatment for halitosis do not actually have genuine halitosis [36]. The presence of taste and smell dysfunction has been suggested as an alternative explanation for halitosis [36], which might also be the case in the patient groups in this study.

The main limitation of this study is the small sample size, especially for non-SS patients. The prevalence of SS has been reported to be 0.05% in the Norwegian population [12]. The low prevalence of SS is also reflected in our study with low sample sizes of both patients with pSS and non-SS. For reasons not clear to us, non-SS patients were more difficult to recruit to the study than patients with pSS. Another limitation of this study is the lack of assessments of dietary intake and body composition of the participants. We continue the inclusion of patients in these categories in our studies at the Dry Mouth Clinic and plan to introduce more dietary assessments in the future.

## 5. Conclusions

In conclusion, this study demonstrated significantly high occurrence of dysgeusia, burning mouth sensation, halitosis, reduced taste, and mouth dryness in non-SS patients and patients with pSS. Impaired smell function and caries experience were more severe in patients with pSS than non-SS patients. Associations were found between participants’ self-reported dental health status and general health status indicating a clear synergy between oral and general health.

## Figures and Tables

**Table 1:** Participant characteristics.

	Non-SS (*N* = 22)	pSS (*N* = 58)	Controls (*N* = 57)	*p*-Value
Age (year)				
Mean ± SD	52.0 ± 10.4	52.9 ± 13.4	49.7 ± 16.5	NS
Range	34–76	26–75	20–79	
Gender				
Female % (*N)*	100 (22)	96.5 (56)	73.7 (42)	<0.001
Male % (*N)*	0	3.5 (2)	26.3 (15)	
Ethnicity				
Caucasian % (*N)*	90.9 (20)	98.3 (57)	93.0 (53)	NS
Non-Caucasian % (*N)*	9.1 (2)	1.7 (1)	7 (4)	
Height (cm)				
Mean ± SD	166.7 ± 5.3	169.5 ± 7.1	170.9 ± 7.1	0.049
Range	158–178	153–190	157–187	
Weight (kg)				
Mean ± SD	72.3 ± 16.3	71.6 ± 13.8	69.1 ± 11.7	NS
Range	51–120	49–120	50–90	

non-SS: non-Sjögren’s sicca patients, pSS: primary Sjögren’s syndrome patients; Fischer’s-exact test, One-Way ANOVA, NS = Not Significant.

**Table 2:** Questionnaire used to assess participants’ complaints of dysgeusia, burning mouth sensation, and halitosis, and their impact on quality of life.

Dysgeusia						
1. Do you experience bad taste on the tongue?	Yes					No
2. If yes, can you describe the taste?	Metallic	Sour	Rotten	Bitter	Other	
3. How often do you experience bad taste?	Constantly	Daily	Sometimes	In bad periods *	Other	
4. Is the bad taste related to meals?	During meals	In between meals	Constantly			
**Burning Mouth Sensation**						
5. Do you experience burning mouth sensation?	Yes					No
6. Where in your mouth do you experience burning sensation?	Whole tongue	Anterior tongue	Lips	Palate	Other	
7. How often do you experience burning sensation?	Constantly	Daily	Sometimes	In bad periods *	Other	
8. Is the burning sensation related to meals?	During meals	In between meals	Constantly			
9. Do you have to refrain from certain food items due to burning sensation?	Yes					No
10. If yes, what kind of food items do you have to avoid?	Spicy	Sweet	Sour	Salty	Bitter	
**Halitosis**						
11. Do you have complaints of bad breath?	Yes					No
12. How often do you have these complaints?	Constantly	Daily	Sometimes	In bad periods *	Other	
**Quality of Life (QoL)**						
13. Which of the disturbances have a negative impact on your QoL?	Burning mouth	Reduced taste/smell	Distorted taste	Bad breath	Dry Mouth	

* Bad periods: periods when disease symptoms are more pronounced.

**Table 3:** Overview of self-reported complaints of dysgeusia, burning mouth sensation, and halitosis in the three groups.

	Non-SS (*N* = 22)	pSS (*N* = 58)	Controls (*N* = 57)	*p*-Value
Dysgeusia % (*N*)	77.3 (17)	60.3 (35)	3.5 (2)	<0.001
Burning Mouth Sensation % (*N*)	59.1 (13)	50.0 (29)	3.5 (2)	<0.001
Halitosis % (*N*)	59.1 (13)	37.9 (22)	1.8 (1)	<0.001

non-SS: non-Sjögren’s sicca patients, pSS: primary Sjögren’s patients; Chi-square test.

**Table 4:** Participants experiencing distorted taste.

	Non-SS (*N* = 22)	pSS (*N* = 58)	Controls (*N* = 57)	*p*-Value
Metallic % (*N*)	63.2 (12)	20.4 (10)	0	
Rotten % (*N*)	15.8 (3)	16.3 (8)	0	<0.001
Bitter % (*N*)	0	14.3 (7)	0	
Other % (*N*)	0	12.2 (6)	1.8 (1)	

non-SS: non-Sjögren’s sicca patients, pSS: primary Sjögren’s patients; Fischer’s-exact test.

**Table 5:** Overview showing how often participants experienced distorted taste.

	Non-SS (*N* = 22)	pSS (*N* = 58)	Controls (*N* = 57)	*p*-Value
Constantly % (*N*)	9.1 (2)	10.3 (6)	0	
Daily % (*N*)	9.1 (2)	12.1 (7)	0	<0.001
Sometimes % (*N*)	18.2 (4)	20.7 (12)	1.8 (1)	
In bad periods % (*N*)	31.8 (7)	8.6 (5)	0	

non-SS: non-Sjögren’s sicca patients, pSS: primary Sjögren’s patients; Fischer’s-exact test.

**Table 6:** Participants reporting whether dysgeusia was related to meals.

	Non-SS (*N* = 22)	pSS (*N* = 58)	Controls (*N* = 57)	*p*-Value
During meals % (*N*)	0	6.9 (4)	0	
In between meals % (*N*)	9.1 (2)	13.8 (8)	1.8 (1)	<0.001
Constantly % (*N*)	0	10.3 (6)	0	

non-SS: non-Sjögren’s sicca patients, pSS: primary Sjögren’s patients; Fischer’s-exact test.

**Table 7:** Location of burning mouth sensation experienced in the oral cavity.

	Non-SS (*N* = 22)	pSS (*N* = 58)	Controls (*N* = 57)	*p*-Value
Whole tongue % (*N*)	40.9 (9)	36.2 (21)	1.8 (1)	
Anterior tongue % (*N*)	9.1 (2)	3.4 (2)	0	<0.001
Lips, palate% (*N*)	0	1.7 (1)	0	

non-SS: non-Sjögren’s sicca patients, pSS: primary Sjögren’s patients; Fischer’s-exact test.

**Table 8:** Overview showing how often participants experienced burning mouth sensation.

	Non-SS (*N* = 22)	pSS (*N* = 58)	Controls (*N* = 57)	*p*-Value
Constantly % (*N*)	9.1 (2)	5.2 (3)	0	
Daily % (*N*)	13.6 (3)	10.3 (6)	0	<0.001
Sometimes % (*N*)	4.5 (1)	19.0 (11)	1.8 (1)	
In bad periods % (*N*)	22.7 (5)	3.4 (2)	0	

non-SS: non-Sjögren’s sicca patients, pSS: primary Sjögren’s patients; Fischer’s-exact test.

**Table 9:** Participants reporting whether burning sensation was related to meals.

	Non-SS (*N* = 22)	pSS (*N* = 58)	Controls (*N* = 57)	*p*-Value
During meals % (*N*)	22.7 (5)	25.9 (15)	0	
In between meals % (*N*)	9.1 (2)	1.7 (1)	0	<0.001
Constantly % (*N*)	4.5 (1)	1.7 (1)	0	

non-SS: non-Sjögren’s sicca patients, pSS: primary Sjögren’s patients; Fischer’s-exact test.

**Table 10:** Overview showing how often participants experienced halitosis.

	Non-SS (*N* = 22)	pSS (*N* = 58)	Controls (*N* = 57)	*p*-Value
Constantly % (*N*)	4.5 (1)	8.6 (5)	0	
Daily % (*N*)	18.2 (4)	6.9 (4)	0	
Sometimes % (*N*)	13.6 (3)	8.6 (5)	0	<0.001
In bad periods % (*N*) After meals % (*N*)	13.6 (3) 4.5 (1)	3.4 (2) 1.7 (1)	0 0	

non-SS: non-Sjögren’s sicca patients, pSS: primary Sjögren’s patients; Fischer’s-exact test.

**Table 11:** Percentage of participants with ageusia (no taste perception) and hypogeusia (reduced taste perception) among participants.

	Non-SS (*N* = 22)	pSS (*N* = 58)	Controls (*N* = 57)	*p*-Value
Ageusia % (*N*)	14.3 (3)	15.5 (9)	1.8 (1)	0.031 *
Hypogeusia % (*N*)	9.1 (2)	25.9 (15)	12.3 (7)	0.084

non-SS: non-Sjögren’s sicca patients, pSS: primary Sjögren’s patients; Chi-square test. * *p* < 0.5.

**Table 12:** Correlations between pathologically low UWS/SWS and the three groups.

	Non-SS (*N* = 22)	pSS (*N* = 58)	Controls (*N* = 57)	*p*-Value
UWS (below threshold) % (*N*)	59.1 (13)	74.1 (43)	8.8 (5)	<0.001 ***
SWS (below threshold) % (*N*)	31.8 (7)	65.5 (38)	5.3 (3)	<0.001 ***

non-SS: non-Sjögren’s sicca patients, pSS: primary Sjögren’s patients. UWS: unstimulated whole saliva, below threshold (≤0.1 mL/min); SWS: stimulated whole saliva (≤0.7 mL/min); Chi-square test. *** *p* < 0.001.

**Table 13:** Correlations between UWS and SWS and dysgeusia, burning mouth sensation, halitosis, taste score, DMFT and FT.

	UWS (*N* = 137) *r*	SWS (*N* = 137) *r*
Dysgeusia	−0.37 ***	−0.37 ***
Burning Mouth Sensation	−0.29 ***	−0.39 ***
Halitosis	−0.27 **	−0.27 **
Taste Score	0.21 *	0.21 *
DMFT	−0.30 ***	−0.27 **
FT	−0.26 **	−0.21 **

non-SS: non-Sjögren’s sicca patients, pSS: primary Sjögren’s patients. UWS: unstimulated whole saliva, SWS: stimulated whole saliva, Pearson’s point-biserial correlation coefficient. *** *p* <0.001, ** *p* < 0.01; * *p* < 0.05.
